# Preoperative Prediction and Identification of Extracapsular Extension in Head and Neck Cancer Patients: Progress and Potential

**DOI:** 10.7759/cureus.34769

**Published:** 2023-02-08

**Authors:** William N Duggar, Toms Vengaloor Thomas, Yibin Wang, Abdur Rahman, Haifeng Wang, Paul R Roberts, Linkan Bian, Ronald T Gatewood, Srinivasan Vijayakumar

**Affiliations:** 1 Radiation Oncology, University of Mississippi Medical Center, Jackson, USA; 2 Industrial Systems and Engineering, Mississippi State University, Starkville, USA

**Keywords:** preoperative, artificial intelligence, model explainability, deep learning, head and neck squamous cell carcinoma, extracapsular extension

## Abstract

Background

This study aimed to demonstrate both the potential and development progress in the identification of extracapsular nodal extension in head and neck cancer patients prior to surgery.

Methodology

A deep learning model has been developed utilizing multilayer gradient mapping-guided explainable network architecture involving a volume extractor. In addition, the gradient-weighted class activation mapping approach has been appropriated to generate a heatmap of anatomic regions indicating why the algorithm predicted extension or not.

Results

The prediction model shows excellent performance on the testing dataset with high values of accuracy, the area under the curve, sensitivity, and specificity of 0.926, 0.945, 0.924, and 0.930, respectively. The heatmap results show potential usefulness for some select patients but indicate the need for further training as the results may be misleading for other patients.

Conclusions

This work demonstrates continued progress in the identification of extracapsular nodal extension in diagnostic computed tomography prior to surgery. Continued progress stands to see the obvious potential realized where not only can unnecessary multimodality therapy be avoided but necessary therapy can be guided on a patient-specific level with information that currently is not available until postoperative pathology is complete.

## Introduction

Head and neck cancer (HNC) is a major health issue among smokers; recently, human papillomavirus (HPV)-induced oropharyngeal cancer among non-smokers is also increasing in incidence [1-3]. Surgery, radiotherapy (RT), and chemotherapy are the main modalities of care for HNC [4,5]. A team approach is unavoidable for the best outcomes in HNC [6,7]. In the early stages of HNC, a single modality, either surgery or RT, can lead to remarkably high local control and survival results with excellent quality of life (QOL) outcomes, for example, for T1 vocal cord cancers with RT and T1 cancer of the oral tongue with surgery [8,9]. Some of the worst prognostic factors for HNC are advanced tumor stage (T3-4), lymph node involvement (N2-3), and distant metastatic disease (M+). Within the group of N+ stages, extracapsular extension (ECE) diagnosed by microscopic pathological examination after surgical resection of the lymph nodes is also associated with poor prognosis, requiring more intensive trimodal therapy, for example, surgery followed by chemoradiotherapy [5,10,11]. Single or bimodal therapy carries fewer complications and better ultimate QOL than trimodal therapy [12,13]. In addition, the total package time (TPT) will be less for a more limited use of modalities than the utilization of multiple modalities. TPT is the time taken to complete the overall treatment from the first day of starting any treatment intervention for cancer. Shorter TPT leads to better local and regional long-term disease control as well as survival [14,15].

For example, in oropharyngeal cancer, the main treatment of N1-2 stages, especially for T1-2 primary stages is a surgical intervention called radical neck dissection (RND); more recently, a less morbid procedure, modified RND (MRND). If only microscopic nodes are found after MRND in one to three lymph nodes and the primary (T1-2) cancer is completely resected with negative margins pathologically, no more treatment other than close, periodic follow-up with smoking cessation can lead to very satisfactory local/regional control and survival outcomes [16-19]. Similarly, for certain T1 glottic cancers with N0 status, RT alone would suffice with similar high percentages of successful outcomes [4,5,8].

With the recent utilization of positron emission tomography (PET) in HNC, N0 status can be confidently recognized in this population [20-22]. This often helps in choosing surgery or RT as a single modality of treatment. However, with the current state of knowledge, ECE can be diagnosed only by surgical dissection of the lymph nodes and microscopic pathological examination. When ECE is found after neck dissection, postoperative RT with chemotherapy will be further required to reduce the probability of recurrence and improve survival outcomes [23,24]. The increase to trimodal therapy is associated with an increase in complication rates and a decrease in QOL.

If ECE can be diagnosed with imaging modalities prior to decision-making on the selection of modalities, then the unnecessary addition of modalities can be avoided with many resultant benefits for individual patients and the health systems [24]; for example, the use of fewer modalities and a more appropriate and upfront selection of modalities in individual patients will lead to fewer long-term complications without compromising the local/regional control rates with a higher ultimate QOL; shorter TPTs; and savings to the healthcare system because unnecessary procedures can be avoided.

As previously reported in the literature, ECE is highly correlated with the size of the lymph nodes, but nodes less than 1 cm may also harbor ECE at a rate as high as 25% [25,26]. Although several studies also evaluated the utility of computed tomography (CT) scans in diagnosing ECE, these were largely unsuccessful [27-34]. Due to the inability of diagnostic imaging to predict ECE, the incorporation of ECE into the clinical staging system has been difficult.

In this paper, we show how artificial intelligence and deep learning (DL) can increase the utility of CT scans, which are routinely done as part of staging workup on all HNC patients, as a method to diagnose ECE upfront and accomplish the goals listed above. If successful, our efforts will have practice-changing implications in the management of HNC.

## Materials and methods

Dataset

Preoperative CT scans were collected retrospectively for 130 HNC patients who underwent neck dissection as an initial part of their diagnostic workup. Institutional Review Board was obtained from the University of Mississippi Medical Center (approval number: 2010-0252). All patients were treated at the same academic medical center. See Table 1 for demographic analysis. For each patient, the pathologic classification of ECE presence was recorded as well as the laterality and node level of the identified ECE if provided by pathology. This dataset was then anonymized and coded for use by colleagues at an external research lab.

**Table 1:** Demographics of data used in training and testing. N is the number of samples.

	Training (N = 110)	Test (N = 20)
Males	73%	75%
Females	27%	25%
Age range (year)	34-91	39-71
Median age (year)	58	57
ECE negative	64%	50%
ECE positive	36%	50%

Machine learning architecture and explainability

**Figure 1:**
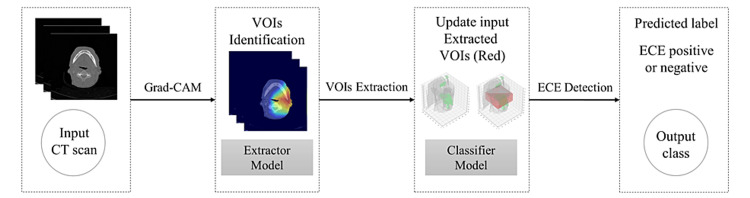
The described GMGENet model framework. VOI = volume of interest; ECE = extracapsular extension; Grad-CAM = gradient-weighted class activation mapping; GMGENet = gradient mapping-guided explainable network

The machine learning architecture utilized in this study is described in great detail in a more technical publication with one exception in that our Hounsfield unit (HU) threshold during training was modified to be -200 to 200 rather than -400 to 400 [35,36]. For a general description, the current model consists of a multilayer gradient mapping-guided explainable network (GMGENet) architecture (Figure 1) involving a volume extractor that defines the volume of interest (VOI) for analysis within the CT dataset and a classifier layer that evaluates voxels within the VOI to produce a patient-level output prediction of the presence of ECE as affirmative or negative.

**Figure 2:**
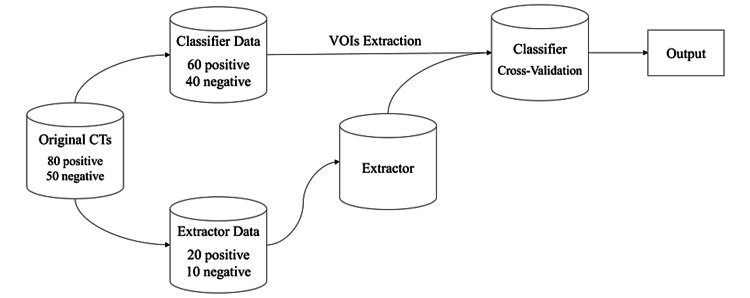
Data flow and sampling in the model development process. VOI = volume of interest

The gradient-weighted class activation mapping (Grad-CAM) approach has been implemented into the algorithm so that not only is a prediction generated regarding ECE presence but the algorithm will also produce a heatmap within the VOI color-coded based on which areas of the CT dataset led the algorithm to decide what the ECE prediction would be for a given patient. The Grad-CAM implementation is also described in more detail in the aforementioned work. The detailed data split for model development is presented in Figure 2. The model has been validated in two aspects, as discussed in the later results section, namely, (1) ECE detection validation, and (2) Grad-CAM explainability validation. Five-fold cross-validation has been applied to avoid overfitting and achieve stable performance during ECE detection validation. Thirty patients were utilized in training the volume extractor layer, and 100 patients were used to cross-validate the classifier, as shown in Figure 2. For the Grad-CAM explainability validation process, 110 patients were used for extractor training, and the remaining 20 patients were held out as a test dataset within which 10 patients were known to be ECE negative and 10 patients were known to be ECE positive, as shown in Table 1. One may note a class imbalance in ECE-positive and negative patients within the training data, but because this imbalance was only slight on the order of roughly 4:6 (four positives for every six negatives), no modifications were made in the handling of this classification problem.

## Results

ECE detection validation performance

Different classification performance measurements are presented, including accuracy, the area under the curve (AUC), sensitivity, and specificity. Accuracy is the proportion of true results, either true positive (TP) or true negative (TN), among the tests. Sensitivity is the proportion of TPs that are correctly identified, which shows how well the test detects ECE. Specificity is the proportion of the TNs correctly identified, which suggests how well the test identifies normal (ECE negative) conditions. These performance metrics can be formulated as:

$Accuracy = \frac{TP+TN}{TP+TN+FP+FN}$

$Sensitivity = \frac{TP}{TP+FN}$

$Specificity = \frac{TN}{TN+FP}$

where TP refers to the number of cases correctly identified as ECE positive, false positive (FP) refers to the number of cases incorrectly identified as ECE positive, TN refers to the number of cases correctly identified as ECE negative, and false negative (FN) refers to the number of cases incorrectly identified as ECE negative.

The results in Table 2 indicate that the model performs at a high level for this dataset, but this prediction is only limited to the general presence of ECE and therefore has limited clinical utility. A clinician would have to make an educated judgment about the actual location of the predicted ECE. Further, a blind prediction may be suspect to a clinician with limited knowledge of how an algorithm provided given results, hence, the need for an explanation of the model’s prediction via the Grad-CAM method.

**Table 2:** Classification performance in the model development process. SD = standard deviation; GMGENet = gradient mapping-guided explainable network; AUC = area under the curve

Performance metric	GMGENet
Accuracy (SD)	0.926 (0.011)
AUC (SD)	0.945 (0.018)
Sensitivity (SD)	0.924 (0.062)
Specificity (SD)	0.930 (0.025)

Grad-CAM extractor explainability validation

Utilizing the Grad-CAM approach, heatmaps were generated for all ECE-positive prediction patients to discern whether the model was identifying clinically relevant areas as part of its decision-making process. Should this prove to be true, then not only would this algorithm be useful for predicting ECE in a given patient but also could provide extremely valuable insight to surgeons and/or radiation oncologists. We validated the interpretability of the proposed Grad-CAM module (GMGENet extractor). Different from the data split of the two-step model framework, our training and test data sampling for clinical validation was based on Table 1. The clinical validation classification performance of the trained extractor is shown in Table 3. Of note, the Grad-CAM extractor is retrained based on the training data, and the data presented in Table 3 are collected based on the test performed on the test data in Table 1. We aim to analyze the extractor explainability given the collected limited samples here.

**Table 3:** Classification performance of Grad-CAM feedback validation. GMGENet = gradient mapping-guided explainable network; Grad-CAM = gradient-weighted class activation mapping

Performance metric	GMGENet extractor
Accuracy	0.85
Sensitivity	0.80
Specificity	0.90
Precision	0.89

**Figure 3:**
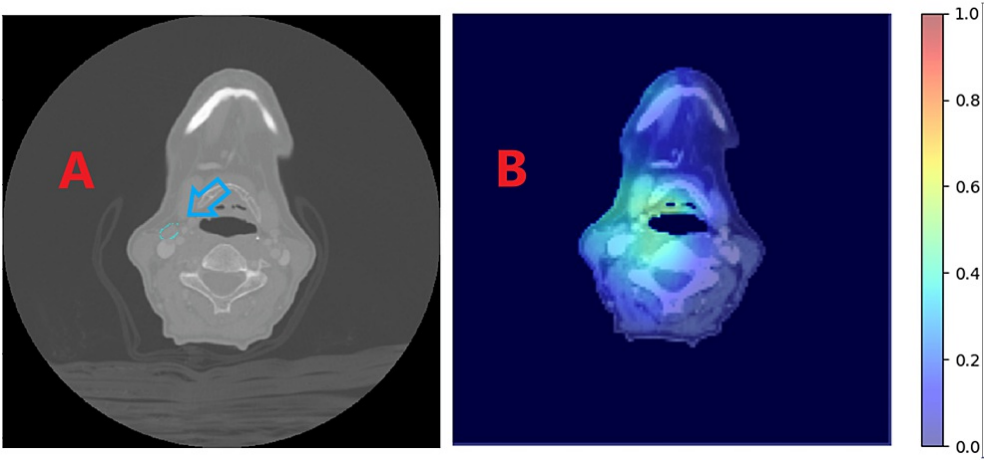
(A) Arrow indicates a lymph node of pathologic significance for ECE. (B) The heatmap generated by the Grad-CAM methodology. Note that the heatmap has highlighted a region adjacent to the indicated lymph node on the same side as the pathologically identified ECE. Grad-CAM = gradient-weighted class activation mapping; ECE = extracapsular extension

**Figure 4:**
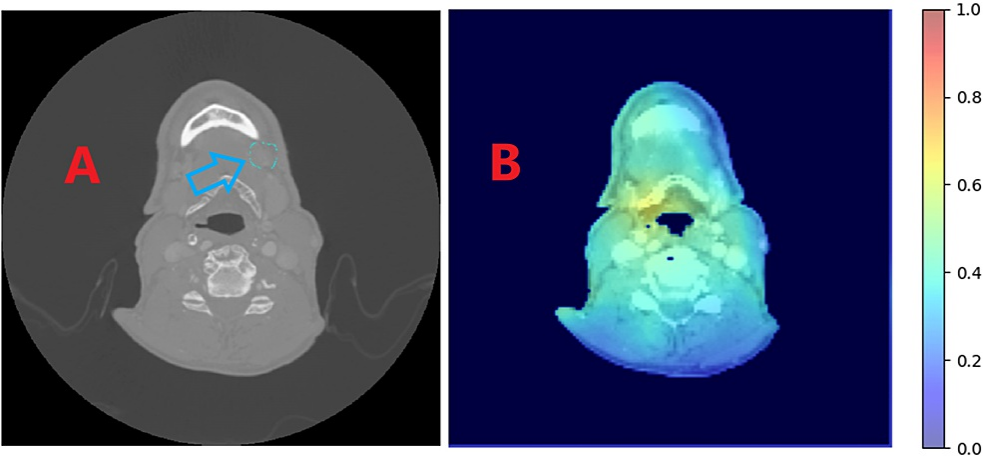
(A) Arrow indicates a lymph node of pathologic significance for ECE. (B) The heatmap generated by the Grad-CAM methodology. Note that the heatmap has highlighted a region opposite to the indicated lymph node of pathologic significance and on the opposite side as the pathologically identified ECE. Grad-CAM = gradient-weighted class activation mapping; ECE = extracapsular extension

The heatmaps generated for the test patients were compared to delineated lymph node contours and pathologic information regarding the laterality of the ECE identified. See Figure 3 for an ideal example and Figure 4 for an example of limited clinical utility beyond ECE prediction. Currently, the model produces mixed results. For some patients, the results are very promising and are clinically reasonable, while, for others, anatomy can be highlighted that is either contrary to the pathologic location of the ECE or meaningless relative to lymph node geography.

## Discussion

Machine learning techniques have been developed over the years to predict ECE from pretreatment imaging. Kann et al. developed a DL algorithm that successfully predicted ECE on pretreatment CT imaging [37]. This algorithm’s performance was then validated using a multi-institutional patient cohort, comparing the model’s performance to board-certified radiologists, and evaluating the benefit of using the algorithm to assist the diagnosticians [38]. Our methodology seeks to build upon this work in that the use of our model does not require lymph node delineation, making its application to the clinic much more practical for radiologists, and the results will be somewhat less of a black box, as areas leading to the conclusion of ECE or not can be highlighted for any reviewing clinician.

While the classification results are quite good, the potential for overfitting and robustness remain a concern. For this reason, future work will seek to increase the variability of the training dataset using more external data. This should increase the algorithm robustness for CTs from various sources and lower the probability of overfitting. Additionally, further testing of this model on external and/or new CT data will offer new insight into the potential improvement of the algorithm against overfitting.

The explainable mechanism of our model has not yet realized its potential, but our belief is that with further training with either additional data or with additional methodology such as adding clinician feedback into the training loop, the advantages of this approach can be realized. We believe this is reasonable and possible because human (radiologist) factors such as increased lymph node size or soft-tissue changes can exist within the CT data leading the clinician to report a higher probability of the presence of ECE. A well-trained DL model should be able to key upon similar features at the very least while also potentially recognizing additional features which may be difficult for humans to observe. These features may be related to less dependence on selected window and level, voxel characteristics, or correlation of certain tumor features (i.e., necrosis), which may not be utilized as predictive factors for a human in the identification of ECE. Additionally, this instance would not be the first for a deep neural network to outperform human observers and be perhaps more robust against uncorrelated noise [39]. Finally, DL studies such as this one can also build trust in proposed intelligent systems by adding explanatory features to the model’s prediction (i.e., heatmaps). Even though some of the current results did not show a clear pattern, it is valuable to track what the DL model deems important which may offer insight and even an opportunity for feedback training to improve.

Should improvement in this mechanism be possible, one can imagine that as the algorithm’s abilities improve and high-risk areas for ECE are highlighted, the clinical practice of oncology among multiple disciplines will be affected. Surgeons will then have pre-pathologic guidance for any operation undertaken which may result in higher efficacy or reduction in toxicity similar to the advent of MRND [16-19]. Should the operation be rejected in favor of other modalities such as chemoradiation therapy, specific target volumes may be generated to also guide such therapy. For example, clinical target volumes are often delineated manually by radiation oncologists to account for microscopic disease, but this process is typically guided by a rough idea of the distance of disease extension into the surrounding tissues and known anatomic boundaries, such as bone. The actual microscopic disease may be either over or underestimated in any given direction. This ECE detection approach would potentially guide the clinician to delineate regions of disease that previously were undetectable to the human eye.

Although the horizon looks promising, the journey is only yet underway. This preliminary work is under review for an NIH R03 grant with the hopes of further training the model by adding clinician-in-the-loop feedback about areas of anatomy that make clinical sense for the algorithm to pay more attention to and others that perhaps are unlikely to carry ECE-relevant information. Additional data from other sources will also be added to training as well to increase robustness and applicability to multiple institutions. In addition to clinician review, the mapping results will be compared with magnetic resonance imaging and PET when possible to further discern the validity of highlighted areas in the Grad-CAM results.

Virtual pathology of lymph node regions such as the methodology above stands to have a major impact on the care and coordination of HNC patients. Once the reliability of ECE detection reaches a certain level, not only physicians but patients may prefer to utilize this information when acquired much less invasively while deciding the optimal course of therapy. Additionally, gains may be realized in the efficiency of care when the time needed for surgery and then pathology is not required for a patient’s treatment course. Here and previously, we demonstrate a budding methodology that can already very reliably detect the overall presence of ECE within a given CT [35,36]. The potential of this technology stands to impact not only the effectiveness of current HNC paradigms but may also adjust them in favor of less toxicity, better QOL, care efficiency, and even healthcare costs.

## Conclusions

With further refinement, this methodology stands to add additional insight and confidence in not only the presence of ECE but also regarding the anatomic region in which ECE is highly probable. One could imagine how useful that may be in defining clinical target volumes during radiation therapy planning. Future work will look to build on the size of the current training dataset with additional CT data from multiple sources and to add physician-in-the-loop methodology into the training process. This will not only reduce the necessary training dataset size but will also add valuable clinician insight into the algorithm’s interpretation of CT data. Once refined, this algorithm will be implemented into phase I clinical trials.

These trials will be two-fold. First, the feasibility of making clinical decisions related to ECE prior to operation will be investigated. Second, the anatomic regions identified by the algorithm as important to ECE will be used to inform target volumes when planning radiotherapy in addition to traditional methods of discerning microscopic disease spread. Indeed, the potential is high for impact on HNC patients.
